# Temporal dynamics of protein complexes in PPI Networks: a case study using yeast cell cycle dynamics

**DOI:** 10.1186/1471-2105-13-S17-S16

**Published:** 2012-12-07

**Authors:** Sriganesh Srihari, Hon Wai Leong

**Affiliations:** 1 Department of Computer Science, National University of Singapore, Singapore 117590; 2 Institute for Molecular Bioscience, The University of Queensland, Brisbane, QLD 4072, Australia

## Abstract

Complexes of physically interacting proteins are one of the fundamental functional units responsible for driving key biological mechanisms within the cell. With the advent of high-throughput techniques, significant amount of protein interaction (PPI) data has been catalogued for organisms such as yeast, which has in turn fueled computational methods for systematic identification and study of protein complexes. However, many complexes are dynamic entities - their subunits are known to assemble at a particular cellular space and time to perform a particular function and disassemble after that - and while current computational analyses have concentrated on studying the dynamics of individual or pairs of proteins in PPI networks, a crucial aspect overlooked is the dynamics of whole complex formations. In this work, using yeast as our model, we incorporate 'time' in the form of cell-cycle phases into the prediction of complexes from PPI networks and study the temporal phenomena of complex assembly and disassembly across phases. We hypothesize that 'staticness' (constitutive expression) of proteins might be related to their temporal "reusability" across complexes, and test this hypothesis using complexes predicted from large-scale PPI networks across the yeast cell cycle phases. Our results hint towards a biological design principle underlying cellular mechanisms - cells maintain generic proteins as 'static' to enable their "reusability" across multiple temporal complexes. We also demonstrate that these findings provide additional support and alternative explanations to findings from existing works on the dynamics in PPI networks.

## Background

Most biological processes within the cell are carried out by proteins that physically interact to form stoichiometrically stable *complexes*. Even in the relatively simple model organism *Saccharomyces cerevisiae *(budding yeast), these complexes are comprised of many subunits that work in a coherent fashion. These complexes interact with individual proteins or other complexes to form functional modules and pathways that drive the cellular machinery. Therefore, a faithful reconstruction of the entire set of complexes (the 'complexosome') from the physical interactions among proteins (the 'interactome') is essential to not only understand complex formations, but also the higher level cellular organization.

Since the advent of "high-throughput" techniques in molecular biology, several screens have been introduced to infer physical interactions among proteins from organisms in a large-scale ("genome-wide") fashion. These have helped to catalogue significant amount of protein interactions in organisms such as yeast, thereby fueling computational techniques to systematically mine and analyse protein complexes from protein interaction (PPI) networks; for a survey of these methods, see [1].

Though these methods have helped to identify a considerable complement of complexes in organisms such as yeast, a crucial aspect overlooked is the 'dynamics' of complexes. Many, if not all, complexes are dynamic entities whose subunits assemble at a particular sub-cellular space and time to perform a particular function and disassemble after that. However, the lack of suitable temporal information (the sub-cellular time at which a pair of proteins interact) in currently available high-throughput interaction datasets makes it difficult to computationally predict and study this dynamic behaviour of complexes. For example, if a subset of proteins in one complex is temporally involved in the formation of another complex but at a different sub-cellular time, then existing complex detection methods working solely on PPI networks cannot disambiguate the two complexes, instead they produce a whole fused cluster of proteins originating from both complexes as a single predicted complex. This severely impacts not only the accuracy of the predictions, but more critically our understanding of the underlying cellular organization. In fact in a recent (2010) foresightful survey by Przytycka et al. [2], the authors emphasize that this lack of temporal information may have led to many cellular processes being wrongly understood. They suggest that if suitable information about the 'timing activities' of proteins can be obtained, the dynamical nature of the underlying organizational principles guiding protein interaction networks and complexes can be better understood.

Towards this direction, several studies have begun on the temporal behaviour of proteins within PPI networks [3-7]. These studies primarily integrate time information in the form of gene expression profiles of proteins with the topological characteristics (positioning of proteins) within PPI networks. These studies have revealed several interesting insights into cellular mechanisms which could not have been understood by ignoring time information, thereby reconfirming the claims of Przytycka et al. [2]. The most important among these findings is the presence of two distinct kinds of 'hub' proteins within PPI networks - 'date hubs' and 'party hubs' - by Han et al. [3].

However, all these works have still only been to the extent of studying temporal behaviour of individual or pairs or small groups of proteins in PPI networks. Since proteins seldom perform their functions in isolation, a deeper understanding of this behaviour can be obtained by studying larger functional groups of proteins. In our work, we study the temporal behaviour of whole protein complexes. We go about doing this by first identifying a suitable "time of reference" onto which the dynamic behaviour of protein subunits within complexes can be mapped, and employ this to study the dynamic assembly and disassembly of whole complexes. We chose the four phases of the yeast cell cycle as this time of reference. Experiments on this reveal an interesting relationship between the 'staticness' of a protein (constant expression across cell cycle phases) and its potential "reusability" across several phase-based complexes - 'static' proteins tend to be highly "reused" across complexes assembled and disassembled during different phases. We suspect that this pattern might be a biological design principle governing underlying cellular functions. Going further, we provide a new classification of proteins based on their temporal participation in complexes, and show that our classification in fact provides additional support and alternative explanations to earlier classifications like the 'date' and 'party' hubs by Han et al. [3].

### A brief survey of works incorporating temporal information into analysis of PPI networks

Most existing works have primarily integrated gene expression profiles with PPI networks to study the relationship between dynamics of proteins and their positioning within networks. Here, we briefly summarize some of these works.

### Correlation between topological positioning of proteins in PPI network and their expression profiles

Based on the analysis using a high-confidence yeast PPI network, Han et al. (2004) [3] reported an interesting dichotomy of hubs in PPI networks - 'date' hubs and 'party' hubs. Both date hubs and party hubs interact with multiple proteins, but date hubs interact with only one protein at a time (context), while party hubs interact with multiple proteins at the same time (context). Han et al. reported a strong correlation between the topological positioning of these hub proteins in PPI networks and their expression profiles - party hubs are 'modular' and are highly co-expressed with their neighbors, while date hubs are 'central' and are not co-expressed with their neighbors. Though this finding was critically questioned by Batada et al. [4,5], the existence of such dichotomy is now increasingly being accepted [6,7], and it paved the way for simultaneous analysis of topologies of networks and their gene expression profiles.

Taking this further, Komurov et al. (2007) [7] studied how proteins with different expression dynamics were positioned in the yeast PPI network. Komurov et al. calculated the statistical expression variance (EV) of each gene in the yeast genome across 272 experiments compiled from SGD [8]. An EV close to 0 indicated a gene with lowest variance (least dynamic), while an EV close to 1 indicated a gene with highest variance (most dynamic). Using a high-confidence PPI network comprising of 5456 interactions among 2315 proteins, Komurov et al. compared the EVs of proteins with their neighbors in the network, and found a strikingly high correlation between EVs of proteins and their neighbor EVs. This suggested that proteins had similar expression dynamics as their immediate neighbors in the network. This confirmed earlier findings (2001) [9] that co-regulated proteins frequently interacted with each other. Carrying this forward, Komurov et al. extended the date-party hub hypothesis of Han et al. [3] by proposing 'family' hubs. Komurov et al. reported that family hubs were constitutively expressed and interacted with their neighbors to form 'static' modules, while party hubs were dynamically co-expressed with their neighbors to form 'dynamic' modules. These static and dynamic modules were enriched with specialized functions.

Yu et al. (2007) [10] studied the topological positioning of hubs in the yeast PPI network, and said 'date' hubs show high betweenness and are therefore inter-modular, while 'party' hubs show high clustering coefficient and therefore intra-modular. More recently (2011), Patil et al. [11] classified hubs in PPI networks using a combination of gene co-expression correlation and co-expression stability among interacting proteins. The co-expression stability measures the extent to which a pair protein is constitutively co-expressed, that is, how "stable" is the co-expression. Based on these two measures, Patil et al. found that hubs showing high co-expression correlation as well as high stability (which they call 'Category 1' hubs) with their neighbors were likely to be intra-modular, while hubs showing low co-expression correlation but high stability ('Category 2' hubs) with their neighbors were likely to be inter-modular. Many of the Category 2 hubs were involved in transient interactions, and corresponded to 'date' hubs.

### The 'dynamics' of complex formation during the yeast cell cycle

de Lichtenberg et al. (2005) [12] studied the dynamics of complex formations during the yeast cell cycle. They constructed a PPI network comprising of 300 proteins (184 dynamic and 116 static) using Y2H and TAP/MS screens. Extraction of complexes from these screens and comparisons with known complexes from MIPS [13] revealed 29 heavily intraconnected modules (complexes or complex variants) that existed at different "time points" during the cell cycle. Further, most complexes contained both constitutively expressed (static) as well as periodically expressed (dynamic) proteins. More interestingly, almost all eukaryotic complexes were *assembled *just-in-time contrary to the just-in-time *synthesis *observed in bacteria. Just-in-time assembly meant that most subunits of complexes were pre-transcribed, while some subunits were transcribed when required to assemble the final complex. This was more advantageous than just-in-time synthesis because only a few components of entire complexes had to be tightly regulated to control the timing of the final complex assembly. Holding off on the last components enabled the cell to prevent "switching on" of complexes at wrong times.

### Our study of protein 'dynamics' in complexes

The discussed works are enough evidence to the claim that understanding of underlying cellular principles can be enhanced by studying the dynamics of proteins together with their topologies in PPI networks. However, these works focus only to the extent of studying pairs of proteins (neighbors) within PPI networks. Since proteins seldom perform their functions in isolation, a deeper understanding can be obtained by studying larger functional groups of proteins in the dynamics context. In our work, we study the dynamics of proteins through their participation in complexes.

### Methodology

Its not straight-forward to study dynamics of whole complexes by directly correlating gene expression profiles of constituent proteins - this involves computing the expression correlations simultaneously among multiple proteins (and not just among pairs) which is not easy. To devise a simpler way, we "discretize" the profiling of proteins so that each protein can be assigned a unique discrete time during which it is active. Essentially, we first choose a suitable 'time of reference' containing *discrete intervals *of time. We then map each protein to a unique interval on this reference based on its peak expression such that two proteins falling within the same interval can be reasonably considered as "co-expressed" or simultaneously active, while those falling within different intervals as "not co-expressed". Once such a profiling of proteins is done, we map all constituent proteins within complexes onto this reference to understand the dynamic behaviour of whole complexes. This makes our analysis simpler as well as insightful, as we shall demonstrate.

Here, we use the *yeast cell cycle *as our discrete time of reference and its *phases *as our intervals. The cell cycle is a highly controlled process for duplication of cells. The yeast (eukaryotic) cell cycle consists of four distinct progressive phases *G*1 (Gap1) → *S *(synthesis) → *G*2 (Gap 2) → *M *(Mitosis). For each protein involved in the yeast cell cycle, we determine the phase in which the protein shows peak expression and map it to that phase. We then study the dynamic behaviour of whole complexes using the peak phases of the constituent proteins.

Of course by adopting only the cell cycle as our time of reference we will be able to study only cell cycle-related complexes. We identified the cell cycle because it is a highly controlled process with distinct temporal phases which makes it easy to bin proteins uniquely into the phases. Secondly, the availability of gene expression data for most of the cell-cycle proteins makes it convenient to compute the phases.

### Experimental set up

We considered the four yeast PPI networks shown in Table 1 for our experiments. All four networks are built from raw TAP-MS interaction data coming from two large-scale screens by Gavin et al. [14] and Krogan et al. [15]. However, datasets produced from large-scale screens are known to contain considerable amount of spurious (false positive) interactions. Therefore, here we first filter the datasets before performing our experiments. We used four reliability scoring schemes, namely, Iterative-CD [16], FS Weight [17], Purification Enrichment (Consolidated network) [18] and Bootstrap-based [19] to score the interactions within the network and filter out the noisy (spurious) interactions. The details of these scoring schemes are detailed in the corresponding references, but to summarize here, these schemes essentially assign a confidence score (range 0 - 1) to each interaction in the PPI network. These scores account for the technical uncertainties in the underlying experiments and therefore reflect the reliability of the interactions. Interactions with scores below a certain threshold (here, we consider 0.20) are discarded, and the remaining interactions are retained for our experiments.

**Table 1:** Yeast PPI networks used in our analysis

PPI Network	# Proteins	# Interactions	Avg node degree
ICD(Gavin+Krogan)	1628	8707	10.69
FSW(Gavin+Krogan)	1628	8688	10.67
Consolidated_3.19_	1622	9704	11.96
Bootstrap_0.094_	2719	10290	7.56

We used four yeast PPI networks derived from the raw datasets of Gavin et al. [14] and Krogan et al. [15] and subsequent scoring using four affinity scoring schemes. The properties of these networks are shown here.

We employed a recent (2010) complex detection method MCL-Caw [20] to predict complexes from the four networks for our study. MCL-Caw clusters the PPI network solely on topological information to identify dense subnetworks, which are output as its predicted complexes. We further used the hand-curated yeast complexes from Wodak CYC2008 [21] to substantiate the findings.

#### Assigning cell cycle phases to proteins

**Figure 1:**
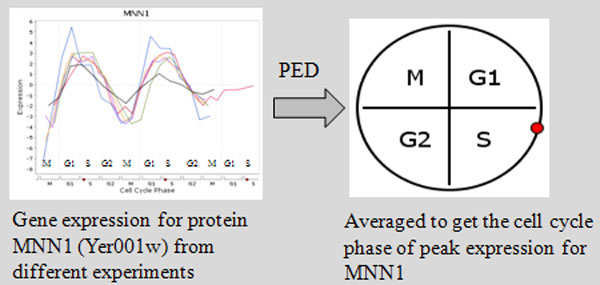
**Protein expression discretization**. For each protein, we calculate the cell cycle phase in which the protein shows peak expression by averaging over multiple gene expression datasets. We call this procedure as Protein Expression Discretization (PED). We take the aid of Cyclebase [22] for this discretization procedure. If a protein is expressed maximum in more than one phase, it is labeled 'static', else it is labeled 'dynamic' along with the phase in which it expresses maximum. Out of the considered 6114 yeast proteins, 5514 were labeled 'static', and the remaining 600 as 'dynamic'. Out of these 'dynamic' proteins, 576 had distinct a peak phase, while the remaining 24 were labeled 'uncertain'.

We assigned a unique cell cycle phase (*G*1, *S, G*2, *M *) to each protein based on the phase in which it showed peak expression. We call this procedure *Peak Expression Discretization *(PED). For computing these phases we took the aid of Cyclebase http://www.cyclebase.org/[22]. Cyclebase averages gene expression datasets obtained from multiple microarray studies to compute the approximate phase of peak expression for each protein (see Figure 1). If a protein is expressed maximum in exactly one phase, it is labeled 'dynamic' along with the corresponding peak phase, else if it expresses maximum in more than one phase it is labeled 'static'. Out of the considered 6114 yeast proteins, 5514 were labeled 'static', and the remaining 600 as 'dynamic'. Out of these 'dynamic' proteins, 576 had distinct a peak phase, while the remaining 24 were labeled 'uncertain'.

### Studying temporal characteristics of PPI networks

To begin with, we integrated the computed cell cycle phases of proteins with our PPI networks and performed an analysis of network dynamics, as shown in Table 2. The table shows that interactions among static proteins (static-static) dominated the network (for example, 94.69% in Consol_3.19_). This is crucial to maintain the stability of the network. The static-dynamic and dynamic-dynamic interactions formed relatively smaller fractions of the networks (for example, S-D: 4.6% and D-D: 0.716% in the Consol_3.19 _network).

**Table 2:** Analysis of 'dynamism' in the four yeast PPI networks

Network	# Proteins		# Interactions				
	Total	Annotated	Total	Annotated	S-S	S-D	D-D
ICD(G+K)	1628	1613	8707	8296	7612	363	42
FSW(G+K)	1628	1613	8688	8296	7612	363	42
Consol_3.19_	1622	1613	9704	8941	8466	411	64
Boot_0.094_	2719	2142	10290	9723	8997	518	79

Here, "annotated" refers to labeled as 'static' or 'dynamic' after the protein expression discretization procedure of Cyclebase [22].

Further, we noticed that some of the dynamic partners of static proteins peaked in different cell cycle phases. In other words, a single static protein was involved in transient interactions with dynamic proteins peaking in different phases. These static proteins were enriched with a variety of Gene Ontology (GO) terms, the prominent ones being signal transduction and transcription. This indicated that these were likely "multipurpose" in nature. Their positioning in PPI networks showed that many of these static proteins were connected to different functional regions and they formed hubs in the networks. This indicated that 'staticness' or constitutive expression of a protein might be linked to the extent of "multipurpose" functions the protein was involved in, and also to the 'central' positioning of the protein in the PPI network.

### Studying dynamics of complexes in PPI networks

**Figure 2:**
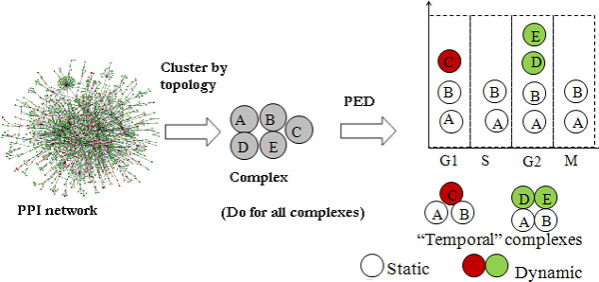
**Workflow for the incorporation of time information into complexes**. We first cluster the PPI network using existing topology-based methods; here we used MCL-CAw [20]. For the clusters that do not match or just partially match known complexes in the Wodak catalogue [21], we incorporate temporal information (in the form of cell cycle phase labels) to check whether the cluster is a fusion of multiple time-based complexes. For this we bin the constituent proteins into the four phases (G1, S, G2, M) based on their phase labels. This procedure helps to decompose large clusters into distinct time-based complexes.

Next, we performed our intended study on protein complexes; the workflow is shown in Figure 2.

#### A case study of cyclin-CDK complexes

**Figure 3:**
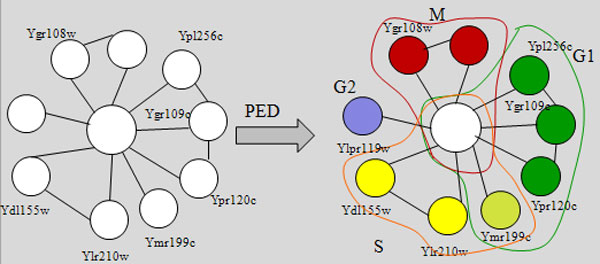
**An example of decomposing a large cluster based on cell cycle phases**. We decomposed a cluster (containing Cdc28) predicted from the PPI network by incorporating cell cycle phase labels of proteins. This helped to identify the different time-based Cdc28-cyclin complexes. These were validated using the Wodak catalogue [21].

Firstly, we present an interesting case study to motivate our analysis. Upon clustering the consolidated net-work using MCL-CAw, we obtained the following cluster containing Cdc28 (Ybr160w): {Ybr160w, Ygr108w, Ypr119w, Ydl155w, Ylr210w, Ypr120c, Ygr109c, Ymr199w, Ypl256c, Yal040c}. When we mapped the cell cycle phase data to the proteins in this cluster, we noticed that the proteins were expressed during different phases: Ybr160w - Static, Ygr108w - *M*, Ypr119w - *G*_2_, Ydl155w - *S*, Ylr210w - *S*, Ypr120c - *G*_1_, Ygr109c - *G*_1_, Ymr199w - G_1_/S, Ypl256c - *G*_1_, and Yal040c - *M *(see Figure 3). This revealed the existence of multiple 'time-based' complexes fused within this large cluster. Therefore, we decomposed the cluster based on the phases into multiple complexes, by assigning the static Ybr160w to each of the complexes. Validation against literature [23] confirmed that Cdc28 (Ybr160w) is a *cyclin-dependent kinase *(CDK) that participates in multiple complexes with its *cyclin *partners, and each of our segregated complexes matched a validated CDK-cyclin complex in the Wodak catalogue [21].

This procedure demonstrated, firstly, how incorporating time information helped to identify time-based complexes accurately which was not possible using only topology information from PPI networks. Secondly and more interestingly, the "reusability" of the 'static' protein Cdc28 across multiple complexes further hinted towards a possible relationship between 'staticness' and participation in multiple complexes or roles.

#### A global study of temporal "resuability" of proteins in complexes

We next performed a large-scale study of all complexes predicted from the yeast PPI networks to further confirm this potential link between 'staticness' and temporal reusability of proteins in complexes. To go about this, we first grouped the proteins within complexes into two sets - the proteins were specialized or unique to complexes, and the proteins that were shared among multiple complexes. We call the specialized proteins as "cores", while the shared proteins as "attachments". If there is a potential link between 'staticness' and temporal reusability of proteins, we expect the attachment proteins to be enriched higher in 'staticness' compared to the cores. We state this as our hypothesis and then test it.

**Hypothesis ***We expect 'staticness' to be more enriched in attachments compared to cores in complexes*.

*Testing our hypothesis: *Let λ_s_(*X*) denote the number of static proteins in set *X*, and *λ_d_*(*X*) denote the number of dynamic proteins in *X*. Using this, we define the *enrichment E *for static (dynamic) proteins among attachments and cores in the set of complexes *C *as follows. For a complex *C *∈ *C *the enrichment in the attachments *Attach*(*C*) is,

(1) $$E_{s}\left(Attach\left(C\right)\right)=\frac{\left|\lambda_{s}\left(Attach\left(C\right)\right)\right|}{\left|\lambda_{s}\left(C\right)\right|},$$

(2) $$E_{d}\left(Attach\left(C\right)\right)=\frac{\left|\lambda_{d}\left(Attach\left(C\right)\right)\right|}{\left|\lambda_{d}\left(C\right)\right|}.$$

Therefore, the *relative enrichment RE*(*Attach*(*C*)) of static to dynamic proteins in the attachments in *C *is,

(3) $$RE\left(Attach\left(C\right)\right)=\frac{E_{s}(Attach\left(C\right)}{E_{d}(Attach\left(C\right)}.$$

**Figure 4:**
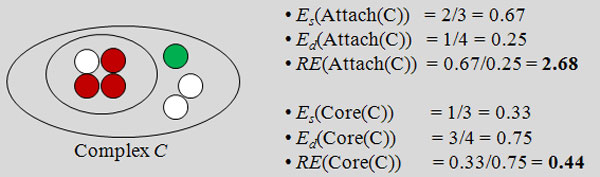
**Calculation of enrichment values for cores and attachments sets of proteins**. An example of how the enrichment values are calculated for cores and attachments. The proteins in inner circle are the cores, while those in outer one are attachments. White proteins are 'static'. In this example, the relative enrichment (static-to-dynamic) of the attachment proteins is 2.68, which is much higher than that of the core proteins (0.44).

The enrichment and relative enrichment for cores is defined in a similar way. See an example calculation in Figure 4. The overall enrichment and relative enrichment for *C *is obtained by averaging over all complexes.

Table 3 shows these values for the complexes predicted from four yeast PPI networks. These values clearly show that the attachment proteins were enriched considerably higher in 'staticness' compared to core proteins, thus supporting our hypothesis. For example, in the Consolidated network, the relative enrichment of 'staticness' for the attachments was *RE*(*Attach*) = 3.402 against *RE*(*Core*) = 0.839 for the cores.

**Table 3:** Analysis of 'dynamism' in cores and attachments of complexes predicted from PPI networks

PPI Network	#Complexes (annotated)		Enrichment *E*		
		Attach		Core	
		**Static**	**Dynamic**	**Static**	**Dynamic**
		
ICD(G+K)	49	0.523	0.179	0.442	0.509
FSW(G+K)	48	0.518	0.177	0.442	0.512
Consol_3.19_	57	0.626	0.184	0.445	0.530
Boot_0.094_	52	0.661	0.192	0.562	0.586

The table compares the relative enrichment of static-to-dynamic proteins within cores and attachments. For complexes (annotated ones only) derived from all four networks, we noticed that the relative enrichment of static proteins within attachments was considerably higher than that of cores. On the other hand, both attachments and cores showed almost equal enrichment of dynamic proteins.

When we mapped some of these complexes back onto the PPI network, we found many of the shared 'static' proteins to be involved in "multiphase" interactions - several dynamic proteins peaking in different phases interacted with these shared 'static' proteins to form dynamic complexes. In other words, the static proteins formed "anchors" for dynamic proteins to form dynamic complexes. These findings hinted towards the biological design principle of temporal "reusability" of 'static' proteins across complexes. The sharing of static proteins among complexes instead of the dynamic proteins ensured maintenance of the generic proteins throughout all phases for their "reusability", while only the dynamic proteins had to be transcribed 'just-in-time' to assemble the required complexes. This strongly agreed with the findings by de Lichtenberg et al. [12]. We analysed some of these shared 'static' proteins and found many to be *kinases *that were involved in activating or deactivating cell cycle complexes. For example, Cdc20 was involved in deactivating the Anaphase Promoting Complex/Cyclosome to allow cell division to enter the *M *phase.

On the other hand, Table 3 also shows that there was no much difference in the enrichments of static and dynamic proteins in the cores, indicating that both static as well as dynamic proteins were equally capable of being part of cores. In other words, specialized sets of proteins may be either static or dynamic. This agreed with the findings by Komurov et al. [7] that both static as well as dynamic proteins were equally capable of forming core functional modules - the static proteins formed 'static modules' while the dynamic proteins formed 'dynamic modules', both of which were involved in vital functions of the cell.

### Relating our findings to previous studies

Based on the analyses here, we relate our findings to previously discussed studies on combining PPI network and gene expression data by Han et al. [3], Kumorov and White [7], Yu et al. [10] and Patil et al. [11], and the work on essential proteins by Pereira-Leal et al. [6]. We provide a new classification of proteins based on their participation in complexes into static "reused" and static/dynamic "specialized" (non-resused) proteins. We relate this classification to that of hubs by the previous works, as show in Table 4.

**Table 4:** Relating our findings with those existing works

	Reused	Specialized	Previous works
Static	'Date' hubs	'Family' hubs	Han et al., 2004 [3]; Komurov and White, 2007 [7]
	Inter-modular	Intra-modular	Yu et al., 2007 [10]
	Category 2	Category 1	Patil et al., 2011 [11]
	Essential		Pereira-Leal, 2006 [6]

Dynamic		'Party' hubs	Han et al., 2004 [3]; Komurov and White, 2007 [7]
		Intra-modular	Yu et al., 2007 [10]

Our classification of proteins is based on their participation in complexes into static "reused" and static/dynamic "specialized" proteins We relate this to the classification of hubs from previous works.

The hub proteins that Han et al. and Kumorov and White categorized as 'date' and 'party' hubs correspond to the static reused proteins and the dynamic specialized proteins within complexes, respectively, in our study. The static reused proteins among complexes interact transiently with different sets of proteins to form different temporal complexes (for example, Cdk kinases), and thereby correspond to 'date' hubs. The dynamic proteins get together to form dynamic complexes at a particular time and disintegrate after that; these correspond to the 'party' hubs (for example, dynamic proteins forming the APC/C complex in G1/S phases). The 'family' hubs of Kumorov and White correspond to the static specialized proteins that form static complexes (for example, the ribosomal complexes). Further, the Category 2 and Category 1 hubs of Patil et al.'s studies correspond to our static reused and static specialized proteins, respectively. Relating to Yu et al.'s characterization of hubs into inter-modular and intra-modular, we note that the static reused hubs are shared among complexes and therefore inter-modular, while the static/dynamic specialized hubs are found within complexes and therefore intra-modular. Finally, relating to Pereira-Leal et al.'s findings, we note that many of our reused proteins are involved in multi-purpose roles (example, kinases), which tend to be essential proteins. These relationships are summarized in Table 4. Therefore, our study provides alternative explanations and additional evidence based on temporal participation in complexes to the classification of hubs from previous studies.

## Discussion

All the analyses shown here are based on the yeast cell cycle as the time of reference. We employed the cell cycle because it is a highly controlled process with distinct temporal phases which makes it natural as well as easy to study the dynamic assembly and disassembly of complexes from their constituent proteins distinctly across phases. Secondly, the availability of data through Cyclebase [22] which averages gene expression profiles from multiple experiments to arrive at distinct peak cell cycle phases for proteins.

However, this also means all our observations and findings are based only on cell-cycle complexes. But we believe the methodology we presented here is insightful and can be replicated across other "times of reference" to study complex dynamics in different scenarios. Sequence of controlled cellular events can be good candidates for such times of reference. One example is the process of DNA damage repair, which involves distinct intervals and checkpoints with different proteins and complexes taking part.

Finally, here we define 'static' proteins as those peaking in more than one cell cycle phase, while 'dynamic' as those peaking in exactly one phase. This definition is reasonable for our analysis here because in the context of the cell cycle there are many proteins that are required and therefore active in exactly one phase. For example, the proteins involved in Synthesis (S) are hardly involved in Mitosis (M). Its only the more constitutively expressed proteins like kinases that tend to be active in more than one phase, which can reasonably considered as 'static'. Having said that, other definitions for 'static' and 'dynamic' are worth testing.

## Conclusion

Many complexes are dynamic entities - their subunits are known to assemble at a particular cellular space and time to perform a particular function and disassemble after that - and while current computational analyses have concentrated on studying the dynamics of individual or pairs of proteins in PPI networks, a crucial aspect overlooked is the dynamics of whole complex formations. In this work, using yeast as our model, we incorporated 'time' in the form of cell-cycle phases into the analysis of complexes from PPI networks and studied the temporal phenomena of complex assembly and disassembly across phases. Through this study we observed an interesting relationship between 'staticness' (constitutive expression) of proteins and their "temporal reusuability" across time-based complexes, which is likely a biological design principle underlying cellular mechanisms. Further, we provided a new classification of hubs based on their temporal participation in complexes, and demonstrated that this classification provided additional support and alternative explanations to the classifications from several existing works.

## Competing interests

The authors declare that they have no competing interests.

## Authors' contributions

SS conceived the study, performed the experiments and analysis, and wrote the manuscript. HWL supervised the project and reviewed the manuscript. Both authors have read and approved the manuscript.
